# Molecular Aspects and Therapeutic Implications of Herbal Compounds Targeting Different Types of Cancer

**DOI:** 10.3390/molecules28020750

**Published:** 2023-01-11

**Authors:** Aditi Sharma, Lalit Sharma, Shouvik Kumar Nandy, Nazrana Payal, Shivam Yadav, Celia Vargas-De-La-Cruz, Md. Khalid Anwer, Haroon Khan, Tapan Behl, Simona Gabriela Bungau

**Affiliations:** 1 Department of Pharmacology, School of Pharmaceutical Sciences, Shoolini University, Solan 173229, Himachal Pradesh, India; 2 School of Biotechnology, Shoolini University of Biotechnology and Management Sciences, Solan 173229, Himachal Pradesh, India; 3 Department of Pharmaceutical Sciences, School of Pharmaceutical Sciences, Chhatrapati Shahu ji Maharaj University, Kanpur 208024, Uttar Pradesh, India; 4 Department of Pharmacology, Faculty of Pharmacy and Biochemistry, Bromatology and Toxicology, Universidad Nacional Mayor de San Marcos, Lima 150001, Peru; 5 E-Health Research Center, Universidad de Ciencias y Humanidades, Lima 15001, Peru; 6 Department of Pharmaceutics, College of Pharmacy, Prince Sattam Bin Abdulaziz University, Al-Kharj 11942, Saudi Arabia; 7 Department of Pharmacy, Abdul Wali Khan University, Mardan 23200, Pakistan; 8 School of Health Science and Technology, University of Petroleum and Energy Studies, Dehradun 248007, Uttarakhand, India; 9 Department of Pharmacy, Faculty of Medicine and Pharmacy, University of Oradea, 410028 Oradea, Romania; 10 Doctoral School of Biomedical Sciences, University of Oradea, 410028 Oradea, Romania

**Keywords:** cancer, anticancer therapies, therapeutics, herbal compounds

## Abstract

Due to genetic changes in DNA (deoxyribonucleic acid) sequences, cancer continues to be the second most prevalent cause of death. The traditional target-directed approach, which is confronted with the importance of target function in healthy cells, is one of the most significant challenges in anticancer research. Another problem with cancer cells is that they experience various mutations, changes in gene duplication, and chromosomal abnormalities, all of which have a direct influence on the potency of anticancer drugs at different developmental stages. All of these factors combine to make cancer medication development difficult, with low clinical licensure success rates when compared to other therapy categories. The current review focuses on the pathophysiology and molecular aspects of common cancer types. Currently, the available chemotherapeutic drugs, also known as combination chemotherapy, are associated with numerous adverse effects, resulting in the search for herbal-based alternatives that attenuate resistance due to cancer therapy and exert chemo-protective actions. To provide new insights, this review updated the list of key compounds that may enhance the efficacy of cancer treatment.

## 1. Introduction

Cancer is a serious problem affecting human health and the second most common reason for death throughout human history. In 2014, the pervasiveness of cancer had increased tremendously; approximately 1,665,540 people were suffering from cancer just in the United States alone, and in 2014, 585,720 of them died due to cancer [1]. Unfortunately, this condition manifests itself at the tissue level, making identification and treatment efficacy extremely difficult [2,3]. Men are prone to the highest percentage of cancer in the prostate, while breast cancer cases are likely to arise in females [4]. Blood cancer, as well as tumors of the brain and lymph nodes, account for the largest number of cancers in children [5,6]. Cancer is a disease with abnormal cell division that spreads through the blood and lymph systems. Tobacco use, excessive alcohol and opiate consumption, environmental pollutants, and ultraviolet radiation exposure are the leading causes of cancer. [7,8]. Cancer is divided into several types, such as carcinomas, which can be further divided into (i) adenocarcinomas as well as squamous, basal, and transitional cell carcinomas; (ii) myeloma and lymphoma leukemia; (iii) bone and soft tissue sarcomas; and (iv) cancers of the brain and spinal cord. In the brain, alterations in DNA sequences produce different types of proteins that cause cerebrovascular disease (CVD). Such patients have abnormal central nervous system (CNS) activity with metastasis and hemorrhage in the nerves. Reduction in body weight, recurrent infectious diseases, illness, breathlessness, muscular fatigue, soreness in the bones and joints, exhaustion, lymph node swelling, and night sweats are some of the most prevalent symptoms [9,10]. Chronic lymphocytic leukemia (CLL), chronic myeloid leukemia (CML), acute lymphocytic leukemia (ALL), and acute myeloid leukemia (AML) are some of several other types of leukemia [11,12,13]. The white blood cells (WBC) count in the bone marrow increases more than usual in acute leukemia. The major causes of leukemia are chromosomal translocation, such as changes in DNA sequences and gene expression anomalies. Chemotherapy and radiation are examples of cancer treatment options. Chemotherapy reduces the activity of oncogene BCR-ABL (Breakpoint cluster region-Abelson proto-oncogene) fusion and removes malignant cells and cancer-affected cells are destroyed by radiotherapy. Cancer can be treated with synthetic medications in addition to surgery. Drugs are being developed to combat uncontrolled B cell proliferation [14]. Cancer can be treated with a variety of medications, e.g., abemaciclib, ribociclib, palbociclib, and imatinib [15,16]. These treatment options are expensive as well as having after-effects, including nephrotoxicity, myelosuppression, leukopenia, neutropenia, thrombocytopenia, mouth mucosal membrane inflammation, hemoglobin deficiency, inanition, general ill health with emaciation, loose motions, and nausea [17]. As a result, scientists discovered several natural compounds that may interact with various proteins and target specific pathways to block cancer growth. The natural compounds have no side effects like those of other drugs and the process of isolating chemical compounds from plants is easier than the production of synthetic drugs in laboratories. Some of the phyto compounds are quercetin, andrographolide, 14-deoxyandrographolide, vinblastine, β-elemene, curcumin, berberine, baicalein, etc. These compounds have the potential to directly control tumor formation as well as cell proliferation during uncontrolled cell growth. Curcumin extract from the rhizome of *Curcuma longa* L. plant inhibits transcription factor NF-κB (nuclear factor kappa B), which leads to cell apoptosis. Berberine is one of the famous natural compounds with anticancer properties. It is derived from many plant species, e.g., *Berberis aristata*, *Chinese schneid*, and *Coptis japonica*. It targets AMPK (Adenosine monophosphate activated protein kinase) and mTOR (mammalian or mechanistic target of rapamycin) molecule signaling pathways by modulating NF-κB, MMP-2 (matrix metalloproteinase-2), and MMP-9 (matrix metallopeptidase 9) proteins to regulate tumor formation [18,19,20,21]. This review summaries the potential of all such natural compounds that can be targeted to combat cancer.

## 2. Pathophysiology of Cancer

**Figure 1:**
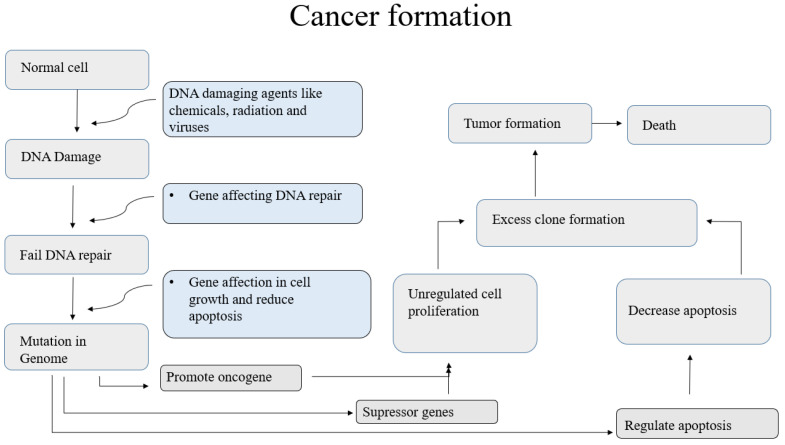
Mechanism of Cancer Formation.

Cancer is the most untreatable disease that results in death worldwide. The main cause of cancer is uncontrolled growth that produces tumors in different organs. Most tumors are caused by genetic changes, gene mutations, DNA damage, or changes in gene sequencing caused by carcinogens. Carcinogenic effects lead to DNA mutations, decreased normal apoptosis, and increased cell duplication, which results in increased cell division (Figure 1). The PI3K/mTOR/AKT (phosphatidylinositol-3 kinase/mammalian or mechanistic target of rapamycin/Ak strain transforming) pathway is the crucial pathway involved in molecular alterations of DNA sequences, including 6% PIK3CA amplification, 7% deletion, and 9% mutation of PTEN (phosphatase and tensin homolog), 4% mutation of PIK3R1, 2% mutation of PPP2R1A and TSC1, 1% deletion of STK11, 3% amplification of RICTOR, and 4% amplification of MTOR. In these pathways, the proteins and RNA become mutated, and the DNA sequence will be copied or deleted. These pathways initiate with the heterodimers of class IA PI3Ks, which also comprise p85 regulatory subunits. After phosphorylation, receptor tyrosine kinases (RTKs) stimulate PI3K via adaptor proteins, such as IRS1 and IRS2. In the amino-terminal domain of PI3K, the adaptor proteins’ interaction results in the inactivation of p110 catalytic subunits, causing inhibition of p85 and p110. The p110 heterodimer converts PIP2 to PIP3 (phosphorylate phosphatidylinositol-4,5-biphosphate) [22]. After the de-phosphorylation, the AKT signaling pathway becomes active. The activation encodes the p85 genes and results in mutation and the activation of PIK3CA oncogenes [23].

**Figure 2:**
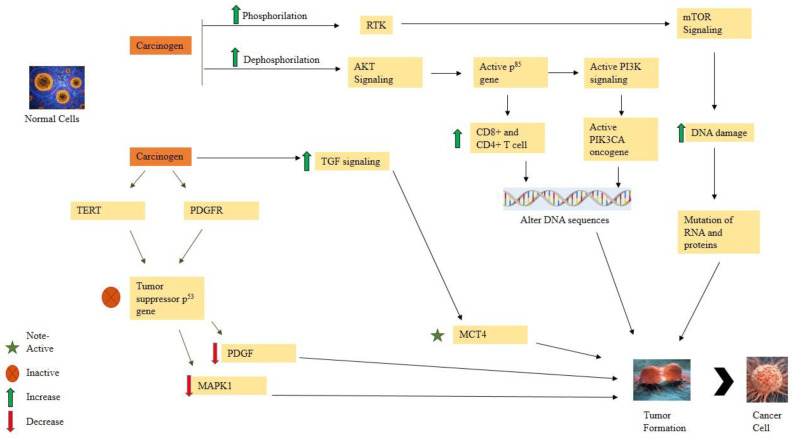
Molecular signaling pathway in cancer formation.

Once the oncogene is turned on, telomerase reverse transcriptase (TERT) and platelet-derived growth factor receptor (PDGFR) get triggered. The reverse transcription decreases MAPK1 (mitogen-activated protein kinase 1) and PDGF (platelet-derived growth factor), which control cell growth. This leads to the rapid growth of the cells and DNA mutations, which inactivate the tumor suppressor p53 gene, further elevating the cell proliferation [24]. Various studies reveal that (FGF19) Fibroblast growth factor 19 amplifies HCC (Hepatocellular carcinoma) in patients. Primarily FGF19 plays a isogenic function with further signaling pathways in liver cancer development like Wnt/β-catenin, epidermal growth factor receptors, the ER (endoplasmic reticulum) related signaling pathway, RAS, IL-6 and STAT3 [25,26]. The chromosomal abnormalities result in a change in the RNA (ribonucleic acid) and ribosomal processing in the cell cycle [27]. Activation of the ERK-MAPK and AKT signaling pathways in cancer patients weakens their immune system by increasing CD8+ T cell proliferation [28]. It also stimulates the CD4+ T cells, NK cells, neutrophils, and G1+ MDSC (Myeloid-Derived Suppressor Cells). As a result, the immune system is harmed in carcinoma [29]. The carcinoma transforms into CAF (carcinoma-associated fibroblasts) after activation of activin A. There is a rise in ROS levels, causing DNA damage. Gene mutation increases IL-6 and VEGF (vascular endothelial growth factor) levels. The CD36 gene is a membrane glycoprotein that presents on the surface of cells and binds fatty acids to facilitate their transport for lipid utilization. Upregulated CD36 expression has been observed in multiple cancer types, including acute myeloid leukemia, colon cancer, breast cancer, gastric cancer, etc. Altered CD36 protein levels lead to the activation of TGF signaling and hypoxia-induced factor-1 signaling, making MCT4 (mono-carboxylate transporter 4) active, which forms tumors. These newly formed tumors are spread by the blood and lymphatic systems. The PI3K/mTOR/AKT pathway play a significant role in changes of DNA sequences, RNA synthesis, and tumor formation [30,31] (Figure 2).

## 3. Types of Cancer and Their Targets for the Treatments

### 3.1. Bladder Cancer

Bladder cancer affects approximately 3.2% of the world’s population, making it the most common type of cancer. The RB (retinoblastoma) pathways and the p53 gene are altered in bladder cancer. With the help of Ras-mitogen-activated protein kinase, these genetic pathways promote proper cell development. When the tyrosine kinase receptor is activated by RAS pathways, HARS (Histidyl-tRNA Synthetase) and FGFR3 (fibroblast growth factor receptor 3) mutations cause tumors. The EGFR (epidermal growth factor receptor) and ERBB2 (erb-b2 receptor tyrosine kinase 2) are both activated by the tyrosine kinase. As a result of the receptors’ overexpression, auto phosphorylation and dimerization occur and the tumor-suppressor gene RASSF1A becomes dormant. The p53 gene turns into the TP53 gene in this malignancy, and p53 governs the cell cycle all over the G1-S conversion. The treatment’s chief goal is to reduce gene mutations by inactivating pathways and boosting the transition of the p53 gene to TP53 [32]. The PI3K/Akt/mechanistic target of rapamycin and receptor tyrosine kinase/related RAS viral (r-ras) oncogene homolog 2 (RRAS2) are the main pathways disrupting cell proliferation and survival. The key signaling pathways implicated in bladder cancer are Ras-MAPK and PKC. To improve therapeutic effectiveness, the EGFR (epidermal growth factor receptors) family (ERBB1, ERBB2, ERBB3, ERBB4) targets are employed. The tyrosine kinase domain of the intracellular EGFR is inhibited by small molecules, and the extracellular ligand-binding area is blocked by monoclonal antibodies [33,34]. Natural compounds, e.g., curcumin, sulforaphane, resveratrol, quercetin, etc., decrease VEGF binding and c-MYC and help to cure bladder cancer [35] (Table 1).

### 3.2. Breast Cancer

The glandular milk ducts’ or breast lobules’ epithelial cells give rise to the malignant tumor known as breast cancer. Around 2.08 million new cases of breast cancer are identified each year in women worldwide. Hormones have a significant influence on the shape and expansion of epithelial tumor cells in breast cancer. Oestradiol (E2) is the most essential regulator of hormonal changes. The aromatase enzyme transforms androgens most efficiently when it interacts with the cellular oestrogen receptor (ER), specifically with the hormones oestradiol, oestrone, and oestrone-sulfate. Stem cell proliferation, differentiation, and motility are regulated using ERs, HER2, and Wnt/ß-catenin, which are the main regulators for healthy breast development and mammary stem cells. Nevertheless, some evidence emphasizes the idea that noncoding RNAs and epigenetic regulators, particularly in triple-negative breast cancer, might serve crucial roles in breast cancer growth. [36]. There are several kinds of oestrogen receptors, the most prominent of which are ER and ERa. According to studies, almost two thirds of breast tumors express too much oestrogen receptor (ER), and more than 70% of them respond to oestrogen-blocking medications such as SERMs (selective oestrogen receptor modulators) and AIs (aromatase inhibitors). SERMs have tissue-specific action and their activity in different cell types and tissues is principally controlled by the recruitment of different cofactors, including co-activators and co-repressors, to ER target genes [37]. Tamoxifen and raloxifene, for example, oppose oestrogen in breast tissue. Tamoxifen, as opposed to raloxifene, behaves as oestrogen within the uterus and has a strong structural resemblance to that of E2, increasing the risk of uterine sarcoma and endometrial cancer [33]. Tamoxifen and raloxifene both raise the risk of thrombosis [38]. The second important molecular target in breast tumors is epidermal growth factor 2 (ERBB2, formerly HER2 or HER2/new), a transmembrane receptor tyrosine kinase of the epidermal growth factor receptor family. Patients with ERBB2 amplified or overexpressed breast cancer are treated with ERBB2-targeted therapies such as anti-ERBB2 antibodies (trastuzumab and pertuzumab) and small-molecule tyrosine kinase inhibitors (lapatinib and neratinib). The abnormal expression of the molecular targets, including ER, PR, or ERBB2, characterizes triple-negative breast cancer, which is found in nearly 15% of breast tumors. The targeted treatment uses drugs to specifically limit the progress of the disease cells without damaging healthy normal cells, in contrast to chemotherapy, which affects both cancerous and healthy cells equally (Table 2). Some of the approved medications to treat breast cancer include letrozole, ado-trastuzumab emtansine, anastrozole, ixabepilone, everolimus, gemcitabine, abemaciclib, fulvestrant, goserelin acetate, doxorubicin hydrochloride, epirubicin hydrochloride, lapatinib ditosylate, and 5-fluorouracil [36,37,38].Curcumin, epigallocatechin gallate, genistein, lycopene, and other natural compounds help in breast cancer treatment by regulating p53 gene expression, upregulating p21, and activating ERK, Akt, and p70S6 kinases [39].

### 3.3. Colorectal Cancer (CRC)

**Figure 3:**
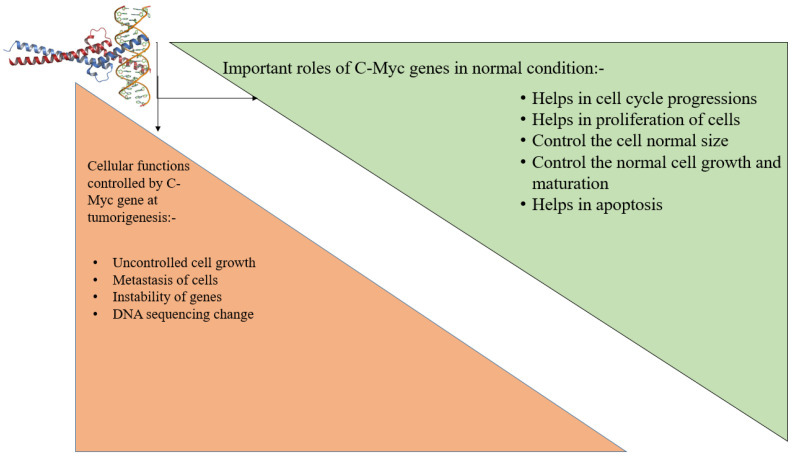
Role of C-Myc gene in normal conditions.

Colorectal cancer is the leading cause of death worldwide. Changes in DNA and RNA characteristics caused by the gene mutation produce an imbalance in a biological process. MSI (microsatellite instability), CIMP (CpG island methylator phenotype), and chromosomal instability are all linked to DNA instability, which causes CRC. In the case of MSI, the repetition of DNA sequences base pair produces gene mutation. The DNA-repairing factor MMR (mismatch repair) (seven mismatched repair genes, such as PMS1, PMS2, MLH1, MLH3, MSH6, MSH3, and MSH2) becomes inactive which results in uncontrolled cell growth [40]. In case of CIN (chromosomal instability), the instability of DNA sequences produces a LOH (loss of heterozygosity), which decreases the allele sequencing and alters the tumor suppressor genes. For example, the p53 gene changes into TP53 in DNA, thus forming a tumor. The target is to stop the DNA mutation, deletion, and instability. Genes like C-Myc have an important role in colorectal cancer. The C-Myc gene normally controls different biological effects. Under normal conditions, C-Myc regulates cell growth, maturation, proliferation, cell cycle progression, cell size, and apoptosis [36]. In this case, the unstable gene is the main cause, but gene mutations (BRAF, RAS, TP53, PI3K, and PTEN) also cause tumor growth in the colon or rectum. The changing lifestyle that leads to tobacco use is an external factor in colorectal cancer [40,41]. When the expression of C-Myc gene increases, the signaling pathways are disturbed. Deregulation causes the cell growth to become uncontrolled, and the genomic changes produce cell metastasis. This gene shows a downstream effect on Wnt-Ras-dependent signaling [42] (Figure 3). Chemotherapies are now often utilized to treat colorectal cancer. The most significant motive of the drugs is to decrease tumor formation and inhibit the signaling molecular pathways that increase rapid cell division. Among the drugs used for colorectal cancer, therapies include aflibercept, bevacizumab, ramucirumab, and regorafenib. A monoclonal antibody called bevacizumab has a strong affinity for vascular endothelial growth factor (VEGF). VEGF binds to VEGF-A receptors and inhibits intracellular signaling pathways, such as KIT, B-RAF, RAF-1, P38 MAPK, and Wnt signaling. The Ramucirumab drug is a member of the Ig G1 class. The main target of this drug is VEGFR-2, which helps to inhibit angiogenesis [43]. Curcumin, a natural compound, inhibits NF-κB, reduces TNF production, and inhibits cancer cell proliferation. Chrysin inhibits p53, p38, MAPK, and apoptosis. Quercetin inhibits cell proliferation by suppressing RASA1 (RAS p21 protein activator) expression [44].

### 3.4. Kidney Cancer

In general nephrology, kidney cancer is a very common disease that is also called renal cell carcinoma (RCC) [45]. In this case, tumor formation occurs in the renal epithelial cells and can be cured by damaging or removing the tumors. Here, the mutation of the gene takes place in the homonymous gene, causing Von Hippel-Lindau (VHL) syndrome. VHL genes encode the protein pVHL. The pVHL protein governs the transcription factor of the hypoxia-inducible factor (HIF). In conditions of low oxygen, the pVHL proteins lead to HIF and transcription [40]. Cell growth, vascular endothelial growth factor (VEGF), platelet-derived growth factor (PDGF), and transforming growth factor (TGF) are enhanced by its transcription. The genes involved in chromatin modulation are KDM5C, BAP1, SETD2, and PBRM1 [46]. The PI3K/mTOR/AKT signaling pathways are also hampered [47,48]. Studies reported that a high level of CD68+ increases tumor-associated macrophages (TAM). VEGF genes amplify cell growth. The main goal of therapeutics is to block VEGF with a monoclonal antibody. Some drugs are given orally to inhibit the VEGFR with kinase inhibitors. Some drugs target the mTOR pathway and control the pathways by introducing serine-threonine kinases [49]. There are different types of chemotherapy used for the treatment of kidney cancer, e.g., sorafenib, sunitinib, pazopanib, tivozanib, cediranib, dovitinib, and regorafenib (Table 3) [50]. Natural compounds such as quercetin reduce lipid ROS and increase glutathione; luteolin increases the p53 gene and decreases PUMA (p53 up-regulated modulator of apoptosis); and kaempferol suppresses TNF and activates NF-κB [51].

### 3.5. Lung Cancer

Lung cancer affects an estimated 1.8 million people annually. Tobacco inhalation, along with passive smoking, is the main cause of lung cancer. Non-small cell lung cancer (NSCLC) and small cell lung cancer (SCLC) are the most common types of lung cancer. The majority of lung cancer patients have NSCLC, whereas the remaining 15% have SCLC. The three kinds of NSCLC recognized by the World Health Organization are adenocarcinoma, large cell carcinoma, and squamous cell carcinoma. In adenocarcinomas, tumors form inside an airway epithelial cell; large cell carcinoma tumors develop in the large nucleoli and cells of the tissue; and squamous cell carcinoma tumors form in the alveolar cell [49]. Lung cancer is associated with mutations in the AKT, ROS, p53, EFGR, MET, and BRAF genes (Table 4). The TKI-sensitizing mutation occurs at the site of exon 19 after tobacco intake. Rapid cell divisions are accelerated in tumors caused by genetic changes and changes in transducing signaling pathways. Several growth factors, including TGF-β, PDGF, FGF, Keratinocyte Growth Factor (KGF), and Hepatocyte Growth Factor (HGF), increase lung abnormalities by causing the unfolded protein response (UPR) [57,58]. The signaling of KGF promotes pulmonary fibrosis. KGF signaling aids in the upregulation of AKT signaling pathways, which in turn aids in the down regulation of PTEN in lung cells via increasing IKK and NF-ΚB. This results in negative signaling, and cell growth accelerates. As a result, excessive KGF secretion causes lung abnormalities and cancer [59]. Excess HGF synthesis raises the amount of Broncho-alveolar lavage fluid in the lungs [60]. AEC2 DNA synthesis is aided by HGH. The AEC2 typically regenerates lung cells that have been destroyed. As a result, numerous factors alter the nucleotide sequence for the AEC2 gene’s production, resulting in lung cancer. For instance, in endoplasmic reticulum stress, there is a high cellular response to protein expression. The UPR increases the number of mediators produced by DNA, which reduces apoptosis. TGF-1 activates the Wnt–Catenin signaling pathway, making apoptosis more difficult to achieve and leading to lung cancer. The medications’ main targets are PDGF, UPR, TGF and TGF, KGF, HGF, FGF, and Wnt–Catenin signaling [61]. Lirilumab, paclitaxel, pembrolizumab, and other medications are available for the treatment of lung cancer [62] (Table 4). Radiation therapy is also used to treat lung cancer. There are primarily two effective forms of radiation. A type of radiotherapy called intensity-modulated radiotherapy (IMRT) uses a solitary radiation beam to treat an afflicted region. The alternate option remains stereotactic body radiotherapy (SBRT), which involves delivering precise light in the direction of the affected region. Radiation aids in the destruction of damaged cells in particular [63]. Curcumin and β-elemene are natural compounds that inhibit the PI3K/Akt pathway and resist the A549/DPP multidrug [64].

### 3.6. Lymphoma

Lymphoma is a cancer of the lymphatic system. Diffuse large B-cell lymphoma (DLBCL) is perhaps the most prevalent lymphoma among the numerous types [68,69]. The lymphoma is produced by the translocation of MYC. The BCL6 gene is a repressor of transcription that works as a regulator of the germinal cellular process. BCL6 (B cell lymphoma 6) regulates B cell receptor activity as well as the expression of CD40 (clusters of differentiation 40) signaling, which activate NF-κB and MAPK signaling pathways. BCL6 is also involved in the regulation of B cell responses to chemokines and cytokines. BCL6 regulates CD8+ T cell activity in an immune response. After introducing the carcinoma factors to the body, the mutation happens at the gene level. For example, the expression of BCL6 becomes deregulated by the mutation of the BCL6 gene, which produces non-Hodgkin lymphomas. The interaction between the domain BCL6 and the POZ/BTB has decreased as a result of the gene mutation. This lack of interaction produces significant toxicity in the body. When the BCL6 levels decrease, the immune system becomes compromised by the inactivation of the T cell immune response. The cell–cell interaction also decreases, which hampers the transcription codon. The main drug targets for lymphoma are BCL6 and the PI3K delta protein [70]. Bleomycin, etoposide, doxorubicin, vincristine etc. are the medications for lymphoma cancer which are commercially available [70,71].

### 3.7. Melanoma Cancer

Melanoma is the most common form of skin cancer and is mainly caused by UV radiation. Every year, approximately 1.7% of new cases of melanoma are diagnosed [72]. In the early stages after sun exposure, the mutation of a normal gene produces an oncogene. The mutation produces the B-Raf proto-oncogene (BRAF) along with the NRAS proto-oncogene (NRAS) [73]. When the skin is exposed to the sun, the gene mutation occurs through the alteration of the gene sequencing and the copying of gene numbers. For example, after sun exposure, C changes to T (UV B) or G changes to T (UV A) [74,75,76]. For the gene mutation, the oncogene activates its signaling and produces BRAFv600, which is formed by the mutation of Val600. After UV radiation on the skin, the normal biological systems will be disturbed, the RAS pathways are activated, and it also activates the MEK1 and MEK2 pathways [77]. These pathways further activate pERK, through which the normal transcription program changes. It can increase cell proliferation and cause changes in glycolysis and mitochondrial metabolism [14]. Aimed at the gene mutation, the tumor protein p53 stimulates mitogen-activated protein kinase (MAPK) pathways. The skin loses its immune system as a result of the gene mutation [78,79]. For the treatment of melanoma cancer, the drug targets will be BRAF and MEK inhibition [71]. Chemotherapy is the most useful treatment for melanoma cancer. Some drugs are PD-1 blocking agents, e.g., Nivolumab and Permbrollizumab, used for melanoma cancer by inhibiting the tyrosine kinase BRAF (Table 5) [80]. The drugs that are much more effective for melanoma treatment are inhibitors of AKT, ERK-1, and ERK-2 activation [81,82]. Natural compounds, e.g., quercetin, kaempferol, and apigenin, inhibit the STAT3 (signal transducer and activator of transcription 3) oncogene and help treat cancer [83].

### 3.8. Oral and Oropharyngeal Cancer

Approximately 4% of all cancer cases are oral cancer, which remains the sixth most frequently diagnosed cancer worldwide. When the cancer is in the oral cavity, it is called “oral cancer”, and if the cancer is inside the throat, it is recognized as “oropharyngeal cancer”. The main risks factors associated with oral cancer are smoking, betel quid chewing, and alcohol intake [84]. The human papillomavirus (HPV) causes throat cancer, and this virus can be transmitted through sex. In oral cancer, malignant tumors form in the oral mucosal epithelium. When the HPV virus enters the body, it represses the TSS (transcriptional start site) with methylation and increases tumor-specific gene silencing [75]. HPV promotes endothelial cell proliferation, cytoskeletal reorganization, NF-κB activation, and inhibits cell apoptosis. T4SS promotes the proliferation of cells and triggers NF-κB signaling which promotes gene mutation. In other cases, the bacteria also secrete a toxin substance, e.g., cytolethal distending toxin (CDT), colibactin, and cytotoxic necrotizing factor 1, increasing carcinogenicity via genomic instability, decreasing DNA impairment response, and causing cell cycle arrest during G2 or M phase [85]. The drugs for oral and oropharyngeal cancer therapy are cisplatin, carboplatin, 5-fluorouracil (5-FU), docetaxel, paclitaxel, hydroxyurea, etc. [86,87].

### 3.9. Pancreatic Cancer

Pancreatic cancer is the most frequently diagnosed cancer globally. Pancreatic cancer patients have a very high fatality rate. In pancreatic cancer, MUC4 (membrane mucin) is among the overexpressed membrane-bound mucins. The MUC4 formation produces neoplastic transformation and tumorigenesis. MUC4 also moderates the interaction of tumor cells with normal cells [80]. MUC4 aids in the metastasis-promoting factor Galectin-3, which aids in gene mutation. In this case, the gene sequencing duplication in allelic produces a variable number of tandem repeat (VNTR) domains in cells. As a result, the cell cycle becomes uncontrollable, accelerates, and results in tumors in target cells. Hence, MUC4, the human mucin gene, has been targeted to treat pancreatic cancer. The inhibition of MUC4 by the RNAs produces a pharmacological effect in pancreatic cancer [88]. Other drugs that are used for this cancer treatment are capecitabine, erlotinib, fluorouracil, gemcitabine, leucovorin, etc. [89].

### 3.10. Prostate Cancer

Prostate cancer is a common type of cancer. Specifically, the secretion fluid, which protects sperm during reproduction, feeds and breeds prostate cancer. There are different signaling pathways, e.g., PI3K, PTEN, AKT, and mTOR signaling, that control the normal cell cycle, growth, as well as its proliferation [90]. Under normal conditions, the PI3K sends a signal to IRS-1 via positive feedback; however, in abnormal circumstances, negative feedback increases protein synthesis as well as cell growth. The drug’s main targets are PI3K, AKT, PTEN, and mTOR signaling [91]. The progression of prostate cancer is significantly influenced by androgen. Androgen produces some enzymes that produce cancer; the glycosylation enzymes are GALNT7, GCNT1, UAP1, ST6GalNAc1, ST6GAL1, CSGALNACT1, and EDEM3. The androgen receptor (AR) promotes gene mutation and amplification by activating these enzymes. The chief targets for prostate cancer are eight main enzymes, GALNT7, GCNT1, UAP1, CYP3A4, CYP2C9, ST6GalNAc1, ST6GAL1, CSGALNACT1, and EDEM3 [92]. Radiation therapy is currently being used to treat prostate cancer. Radiotherapy can be used to destroy specific tumor cells, but there is a slight risk of causing negative feedback to neighboring cells. One of the best radiotherapies is brachytherapy. In brachytherapy, iodine, iridium, cesium, and palladium types of radioactive material are inserted into the body to help cure prostate cancer [93]. Bicalutamide, Abiraterone, Enzalutamide, and other drugs are used to treat prostate cancer [94,95] (Table 6). The natural compound berberine is reported to promote p53 genes and ROS production and can be used to treat cancer. It also helps in paraptotis, particularly by causing mitochondrial swelling and ER dilatation [96].

### 3.11. Thyroid Cancer

In thyroid cancer, the tumor forms in the thyroid gland, commonly known as papillary thyroid cancer (PTC). It is formed by PCT-RAS-BRAF molecular signaling. The BRAF gene mutation is most common in PTC [100]. Studies reported that the deregulation of microRNAs (miR-146b) produces the PTC in several ways. In the case of PTC, the genetic rearrangement in the RET/PTC and NTRK pathways produces tumors by activating the MAPK pathway. MAPK signaling mutates the BRAFV600E gene, which produces metastasis of lymph nodes and tumors. The miR-146a and miR-146b are post-transcriptional gene silencers that play an important role in the immune system [100]. For the gene mutation, the codon gene sequencing is changed. For that, miR-146a and miR-146b are changed into MIR146A and MIR146B. As a result, the chromosomal sequence changes and increases NF-κB signaling. This signaling helps to increase platelet-derived growth factor (PDGF), which increases cancer cell size with the help of growth factor receptor signaling. The Wnt/-catenin pathways are activated after genetic changes by modulating the epithelial-mesenchymal transition (EMT). Negative regulation of the miR-146b gene results in HDAC3 increasing and producing radioactive iodide sensitivity in thyroid cancer cells. As a result, the p21 gene produces a protein that controls tumor proliferation. Following numerous studies, researchers conclude that the main drug target for treating thyroid cancer is the PTC-RAS-BRAF/RET signaling pathways [101]. Drugs that are commercialized to treat thyroid cancer are Axitinib, Lenvatinib, Cabozantinib, etc. [100] (Table 7).

### 3.12. Uterine Cancer

Uterine cancer is one of the most common cancer types in women of reproductive age. The formation of tumors in the uterus produces uterine cancer. In the case of lymphoma, the extracellular matrix (ECM) protein level increases, increasing Rho/ERK/p38 MAPK signaling. The ECM protein regulates cytokines and steroid hormones. In this case, TGF-s and activin-A play an important role in myofibroblasts’ production and promote fibrosis. ECM activation activates FAK (focal adhesion kinase) polymerization and AKAPI3, Rho A, which interacts with ROCK (Rho-associated kinase) receptors along with stimulating ERK and p38 MAPK signaling. The tumor formation in the uterus during reproductive age produces chronic inflammation. In that case, surgery is the first choice, but there is a risk because surgery can produce a negative effect on cell proliferation as well as gene mutation. As a result, primary drug targets are used to inhibit TGF-signaling and TGF-signaling [104].

### 3.13. Adenoid Cystic Carcinoma (ACC)

Adenoid cystic carcinoma happens to be an uncommon cancer with a slow pace of progression. This type of tumor has been discovered in approximately 1% of all cancers worldwide. This type of cancer affects the salivary glands of the head and neck region, tongue, palate, lacrimal gland, and so on. This type of tumor forms in epithelial cells [105]. These types of cancer form by the mutation or deletion of homozygous F-Box and the repeat domain of WD, comprising seven genes (FBXW7). The carcinoma cells are specially divided into two types: one is myoepithelial cells that are present in the periphery of tubules in a proper arrangement, and the other is ductal cells that are present in a pseudo-lumens way [106]. This type of cancer also depends on the mutation of the PTEN, FGFR4, FGF16, and 1LR17RD genes [107,108]. These tumors form via two main pathways, one of which is the activation of the Wnt/-catenin signaling pathway, which increases the transcription level factor Sox4 in RNA and results in tumor formation. The other pathway is the high level of tyrosine kinase c-KIT, whose overexpression increases FGFR1 (fibroblast growth factor receptor 1), HER2, and EGFR. They aid in the deletion of specific chromosomal sequencing, resulting in the production of MYB oncoproteins and a decrease in cell apoptosis [107]. Some of the apoptosis markers are BCL2, API5, BIRC3, and SET. These markers also increase the PI3K pathways for tumor production. So, the main drug target to treat this kind of cancer is the inhibition of the MYB oncoprotein [105]. Drugs, e.g., Axitinib, Dovitinib, and Nelfinavir, are used to treat this type of cancer (Table 8) [109,110].

### 3.14. Amyloidosis Cancer

Amyloidosis cancer is among the rarest cancers in which the clonal disorder is shown in plasma cells. Here, the decreasing number of B cells produces organ dysfunction. The CD38 gene is activated and binds to daratumumab. After binding its Fc fragments, it produces a natural killer cell, damaging normal cells. The main target to treat this cancer is the CD38 gene [112]. This type of cancer is produced by genetic disorders, e.g., a mutation of gelsolin, apolipoprotein, and transthyretin (TTR). For that reason, tumors in the heart, kidney, nerves, and gastrointestinal tract also produce insoluble amyloid fibrils [113]. The drugs are targeted to inhibit the transcription of the TTR genes. This disease can be determined by measuring the level of monoclonal protein (MP) in serum and urine. Thalidomide, in combination with dexamethasone and cyclophosphamide, is the most commonly used drug. It aids in healing by inhibiting toxicity in the body. Other medications, e.g., lenalidomide and melphalan, are used to treat this form of cancer [114].

### 3.15. Anal Cancer

It is an extremely rare kind of cancer that mostly affects women. Each year, around 0.5 percent of cancer cases are of this kind. The HPV (Human papillomavirus) virus is mostly responsible for this form of cancer [115]. The HPV virus induces cancer in the cells of the squamous tissues. The major origin of cancer is the HPV virus transmitted during sexual contact. The cancer causes include tobacco inhalation, aging, and immunological suppression in HIV/AIDS patients. HPV virus contains E6 and E7 viral proteins as well as dsDNA. Viral protein E6 attaches to the tumor suppressor p53 in the cell, causing p53 to be damaged and cell proliferation to rise. Retinoblastoma attaches to E7 which prevents apoptosis, resulting in an unregulated cell cycle [116]. Anal cancer is caused by mutations in the TP53, PIK3CA, and FBXW1 genes, as well as MYC, RICTOR, SOX2 gene amplification, and PTEN gene deletion [117]. Fluorouracil is used to treat anal cancer. Mitomycin C or cisplatin is frequently combined with fluorouracil for anal cancer treatment [118]. Similarly, radiotherapy can be utilized to treat anal cancer since it aids in the destruction of particular tumor cells in specific regions [119].

### 3.16. Astrocytoma Cancer

Astrocytoma is a rare form of cancer that develops on its own. It is most commonly observed in children. The gene mutation in the cerebrospinal fluid is caused by NF2 (neurofibromatosis type 2). The SCH (schwannoma) gene is encoded by the NF2 gene, which causes mutations in the SMARCB1 gene and causes tumors. The MRI is used to diagnose this type of cancer [120]. The major goal is to inhibit the production of NF2 genes and prevent NF2 genes from causing gene mutations. Astrocytoma cancer is treated with medications, such as afinitor, everolimus, bevacizumab, carmustine, temodar, temozolomide, avastin, lomustine, etc. [121,122].

### 3.17. Bone Cancer

Bone cancer is becoming a more common malignancy. When the bone is destroyed, sufferers experience discomfort. An increase in gene mutations activates the mTOR pathway. The mTOR pathway regulates the phosphorylation of 4E-binding proteins. The phosphatidynositide 3-kinase transforms the p70 ribosomal S6 protein kinase (p-PI3K). A bone tumor results from an increase in protein kinase caused by PI3K-L signaling [123]. Sclerostin is another bone cancer route. It primarily acts by enhancing the connections between tumors and normal cells. It promotes the generation of cytokines that inhibit bone resorption. Sclerostin raises RANKL (Receptor activator of nuclear factor κB), resulting in an increase in osteoclasts and a reduction in bone remodeling [124]. Sclerostin and the mTOR pathways are the key therapeutic targets.

## 4. Natural Compounds with Anti-Cancer Properties

### 4.1. Quercetin

**Figure 4:**
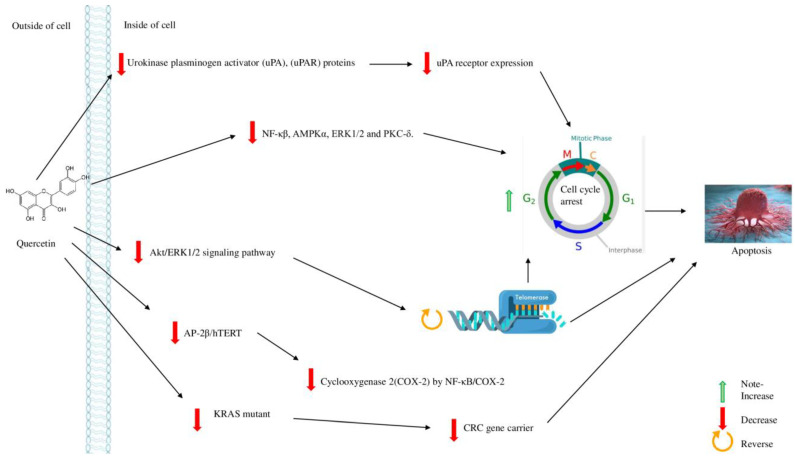
Pharmacological effects of Quercetin in cancer.

Natural compounds, including flavonoids and polyphenols, are reported as anti-cancer agents and can be used against various kinds of cancer (Figure 4). In this regard, quercetin can be broadly observed in daily foods including teas, vegetables, nuts, and different plants. Orally, 1 g of quercetin per day is safe and sufficient, with up to 60% absorption. Based on IUPAC, it is known as 3,3′,4′,5,7-pentahydroxy flavone. Quercetin primarily inhibited gastro-intestinal cell migration, viability, and invasion by lowering urokinase uPA (plasminogen activator), uPAR proteins, and uPA receptor expression, all of which are strongly linked to GC metastasis [125]. Quercetin works as an anti-metastatic agent against GC metastasis cells by interfering with the uPA/uPAR systems, NF, AMPK (Adenosine monophosphate-activated protein kinase), ERK1/2, and PKC (Protein kinase C). Quercetin’s effects on colorectal cancer (CRC) cells carrying the KRAS mutant gene revealed that it might increase apoptosis in cancer. It inhibits proliferation of ARP-1, MM.1R, and RPMI8226 in several myeloma cell lines by inducing apoptosis as well as cell cycle arrest in the G2/M phase. It repressed the Akt/ERK1/2 signaling pathway, hTERT (telomerase reverse transcriptase) through impending AP-2/hTERT, and COX-2 (cyclooxygenase 2) by inactivating NF-κB and COX-2. It targets AMPK α, PI3K-Akt, Akt/ ERK ½, NF-κβ, COX-2, ARP-1, and RPMI8226 to show anticancer property (Table 9) [125].

### 4.2. Andrographolide and 14-Deoxyandrographolide

**Figure 5:**
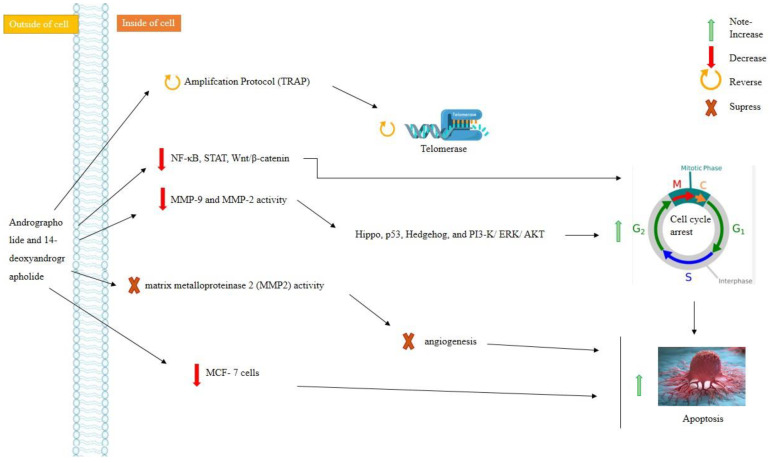
Andrographolide and 14-deoxyandrographolide in cancer treatment.

Andrographolide and 14-deoxyandrographolide are derived from *Andrographis paniculata*, family Aceanthaceae. They are reported to have anti-cancerous properties. Ethanol, dichloromethane, methanol, and water extracts of this plant contain andrographolide and 14-deoxyandrographolide that work against cancer via telomerase inhibition (Figure 5). They inhibit MCF-7 cells’ TRAP (telomerase using the repeated amplification protocol). Studies showed that they inhibited telomerase by 73.7 ± 1.81%, 77.5 ± 1.81%, 78.5 ± 1.35%, and 80.3 ± 1.4%, respectively [126]. Furthermore, the flow cytometry analysis revealed that the methanol and water extracts induced higher rates of total apoptosis by 25% and 32.8%, respectively, compared with 10.07% dichloromethane and 10.7% ethanolic extracts [116]. Anticancer drug development strategies nowadays focus not only on classical pathways, but also on specific signaling pathways, such as NF-κB, STAT, Wnt/-catenin, Hippo, p53, Hedgehog, and PI3-K/ERK/AKT. Andrographolide is inactive for ERK and AKT signaling, down-regulates the PI3-K pathway, and inhibits MMP2 (matrix metalloproteinase 2) activity, which is a regulatory cancer pathway. They also dominate MAPK signaling, the tight junction pathway, and focal adhesion to increase apoptosis. They are responsible for inhibiting cell proliferation, migration, and invasion, and consequently dominant MMP-7 expression, MMP-9, and MMP-2 activity, as shown in Figure 5 [127].

### 4.3. Vinblastine

**Figure 6:**
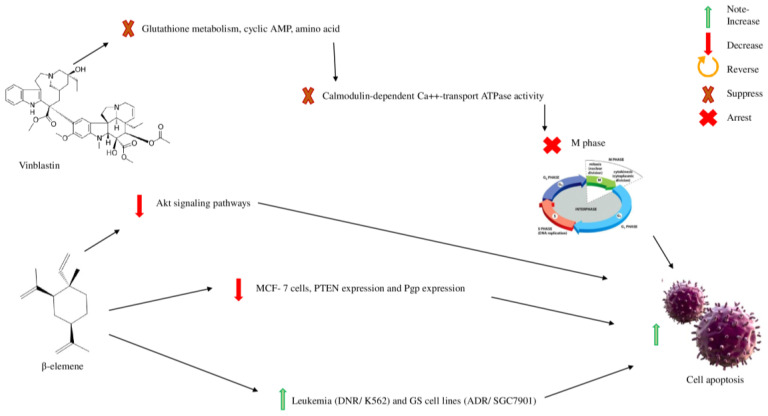
Vinblastine and β-elemene in cancer treatment.

Vinblastine is a semi-synthetic alkaloid derived from the plant *Catharanthus roseus*, family Apocynaceae. This plant extract was administered to rabbits to discover the plant’s anti-diabetic function which revealed its potential as a chemotherapeutic agent (Figure 6). In the trial, rabbits died of bacterial infection due to a lack of WBC, and vinblastine was thought to be helpful against malignancies of the WBC, e.g., lymphoma. Vinblastine prevents microtubule formation, which causes cells to enter the M phase. Biomolecules, e.g., glutathione metabolism, cyclic AMP, amino acids, calmodulin-dependent Ca++ transport ATPase activity, cellular respiration, and lipid biosynthesis, are affected by this compound. Vinblastine may be used to treat non-Hodgkin lymphomas, Hodgkin’s lymphomas, breast cancer, renal cell carcinoma, Kaposi sarcoma, and testicular cancer. Due to adverse reactions, it causes typical side effects, such as myelosuppression, fever, anemia, alopecia, and mucositis (Figure 6) [128].

### 4.4. β-Elemene

β-elemene, a sesquiterpene natural compound, is used in traditional Chinese medicine to treat numerous types of cancer without any severe adverse effects. It increases the sensitivity of multidrug-resistant leukaemia (DNR/K562) and GS (glutamine synthetase) cell lines (ADR/SGC7901), downregulates Akt signaling pathways, and blocks the ABCB1 transporter efflux portion, which is overexpressed in KB-C2 cells. β-Elemene inhibits MCF-7 cells, increases PTEN expression, and increases Pgp expression to increase cell apoptosis, which aids in cancer treatment (Figure 6). It targets DNR/K562, GS cell lines, ABCB1 transporters efflux portion, and MCF-7 to show anti-cancer property (Table 9) [129].

### 4.5. Curcumin

Curcumin is an anticancer natural compound derived from the rhizome of the *Curcuma longa* plant, which belongs to the Zingiberaceae family. By inhibiting AP-1 and hypoxia-inducible factors, it promotes hypoxic stress and the activation of beta-growth factor (TGF-). HIF-1 promotes the expression of VEGF [130]. In tumors, HIF-1 promotes active angiogenesis. It reduces the expression of vascular cell adhesion molecule-1, intracellular adhesion molecule-1, and E-selectin, all of which play important roles in cellular adhesion. Curcumin inhibits the expression of MMPs, ICAM-1, and VCAM (Vascular Cell Adhesion Molecule), all of which are involved in metastasis and cellular adhesion. It also increases several anti-metastatic proteins, such as the non-metastatic gene NM23, tissue inhibitor metalloproteinase (TIMP 2), and E-cadherin. Curcumin is reported to prevent cancer in several organs (Table 9) [130,131].

### 4.6. Salinosporamide A

Salinosporamide A is a potent proteasome inhibitor being studied as a potential anticancer agent. It is currently being established by Nereus Pharmaceuticals under the name NPI-0052. It is cultivated from the *Salinisporatropica* bacterium. It inhibits NF-κB activation (Table 9). NF-κB plays an important role in producing tumors as well as increasing cell division. Salinosporamide A is used to treat multiple myeloma, lymphomas, and solid tumors [132].

### 4.7. Chalcones

Chalcones are precursors for isoflavonoids and flavonoids. The chalcone family has established potential in vivo and in vitro activity against cancers through numerous mechanisms, including autophagy regulation, cell cycle disruption, apoptosis induction, and inflammatory mediators. It showed potential activity against the HCT116, MCF-7, and 143B cancer in vitro cell lines. Furthermore, it causes cell cycle arrest in the M/G2 phase of the HCC cell line via apoptotic cell death [133].

### 4.8. Baicalein

Baicalein is one of the most important active compounds, isolated from the roots of *Scutellariae radix*, which is commonly known as Chinese Huang Qin. It inhibits Akt phosphorylation and the growth of T24 cells by blocking cell cycle progression at the S/G1 phase of division by activating caspase-3 and caspase-9, upregulating Bax expression, and down regulating Bcl-2 expression. Baicalein increases apoptosis. It also inhibits the growth of bladder cancer by preventing the activation of cyclin B1 and CDC2. It strongly induced ER-positive MCF-7 cell apoptosis by inhibiting 17-estradiol-induced transactivation [134]. It promotes the activity of the ERK/p38 MAPK pathway, which helps to prevent cell proliferation in breast cancer. In colorectal cancer (CRC), baicalein suppresses the activation of MMP-9 and MMP-2 by Akt pathway inhibition. Baicalein is also used to treat gastric cancer. It reduced the expression of molecules associated with metastasis, such as vimentin, N-cadherin, ZEB2, and ZEB1. It activates the Akt, PTEN, and HIF-1 pathways, which help inhibit cell proliferation. Baicalein can cure hepatocellular carcinoma as it helps in cell cycle arrest in the G1 phase. By increasing the Bax/Bcl-2 baicalein ratio, it activates caspase-3 and -9, decreases I-kappa-B (IKB)-phosphorylation, and increases p65 and p50 nuclear translocation [134].

### 4.9. Neferine

Neferine is a type of bisbenzylisoquinolone alkaloids derived from the lotus plant *Nelumbo nucifera* Gaertn. It has anti-hypertensive, anti-pulmonary fibrosis, and many other pharmacological activities. Against cancer, many chemotherapeutic agents are nonselective and have a high mortality rate due to adverse effects, but neferine is a targeted natural compound with fewer side effects [135]. Various studies revealed that neferine-enhanced cisplatin induces autophagic cancer cell death by down regulating the PI3K/Akt/mTOR survival signaling pathway, as well as PI3K/CIII-independent autophagy and ROS-mediated Beclin-1 in human lung A549 adenocarcinoma cells. It suppresses cancer cell proliferation by reducing ROS-mediated mitochondrial dysfunction and active MAPK modulation. Niferine promotes cell apoptosis and inflammation by down regulating Bcl2, cycline D1, NF-κB, PI3K/Akt/mTOR signaling, PRAP cleavage, p21, p27, Atg7, Lc3b, JNK/MAPK protein, and Bcl2 protein [135,136].

### 4.10. 9-Methoxy Ellipticine

The compound 9-methoxy ellipticine is derived from plant *Ochrosia elliptica* leaf extract. The common name for it is Ochrosia, and it belongs to the family Apocynaceae. Studies reported that this phytochemical holds anti-cancer, anti-inflammatory, and antioxidant properties. This phytochemical reduces cancer-causing MCF-7 cells from multiplying. Cdc25 phosphatase, thus, is the key target. The cell proliferation becomes mild, and the tumor formation stops once the Cdc25 phosphatase is inhibited [137].

### 4.11. Rutin, Scopoletin, Kaempferol

The *Morindacitrifolia* fruits, which are members of the Rubiaceae family, provide several compounds, e.g., rutin, scopoletin, and kaempferol. Studies reported that these compounds can be used to treat breast cancer [138]. These natural compounds are reported to lower MDA-MB-231 and MCF-7 cell numbers. A greater amount of cell proliferation becomes moderate as a result of this inhibition, which causes apoptosis in a typical fashion. Rutin, scopoletin, and kaempferol are also reported to control the mitochondrial membrane potential [138].

### 4.12. Ginsenosides

The bioactive triterpenoid ginsenosides is derived from many plants’ rhizomes, mainly *Panax notoginseng* (Burk.), F.H. Chen, *Panax ginseng*, and *Cinnamomum cassia* Presl. This compound has anti-cancer, antioxidant, and anti-inflammatory properties. The main compounds among ginsenosides are ginsenoside Rg3, ginsenoside Rh2, and compound K, which are reported in preventing cancer. Studies reported the inhibition of cell viability and cell apoptosis by ginsenoside Rg3 in human ovarian cancer HO8910 cells [139]. Ginsenoside Rh2 induces cell death by activating p53 in human colorectal cancer cells (HCT-116 and SW-480) [140].

### 4.13. Aloe-Emodin

Aloe-emodin (a bioactive anthraquinone) is present in *Rheum palmatum*’s rhizome. This compound triggers apoptosis in H460 cells (Table 9). Studies reported that aloe-emodin treatment reduced the expression of hoGG1, hMHT11, and apurinic endonuclease in H460 cells. This natural compound showed positive results in the treatment of human lung cancer [141].

## 5. Conclusions

Cancer is a leading cause of death worldwide. In this disease, the main factor is excess cell growth. Researchers find different types of factors that cause cancer. There is no age limit for the cancer infection, and anyone from an infant to senescence can be affected by cancer. Studies proved that cancer is caused by the alteration of a gene or a genetic mutation. For cancer treatment, many forms of chemotherapy and radiation are commercially available. However, while cancer cannot be completely cured at this time, a person’s life can be extended by taking medications and receiving radiation. By taking medicine, some side effects are shown to the patients, and those treatments are also expensive to the people of rural and developing areas. Researchers reported some natural compounds, e.g., plant extracts, that have anti-cancer activity. This review summarizes numerous natural compounds that are tested for anticancer activity. These natural components can be used in cancer treatment. These elements can regulate cell growth, genetic changes, cell proliferation, gene changes, gene deletions, and excessive protein synthesis.

## Figures and Tables

**Table 1:** Clinical trials and characteristics of anti-cancerous medications [29].

Agent	Features
Bevacizumab (Phase III)	VEGF-A binding/inhibition
Ziv-aflibercept^196^ Phase I	VEGF binding/inhibiting agent
Cabozantinib (Phase II)	VEGFR-2 inhibitor
Pazopanib (Phase II)	inhibitor of several tyrosine kinases
Tamoxifen (Phase II)	An antagonist of estrogen receptors
Buparlisib (Phase II)	Pi3K inhibitor
Dovitinib (Phase II)	FGFR and veGFR inhibitor
MeK162 (Phase II)	MeK inhibitor
MGAH22 (Phase I)	HeR2-targeting antibody
Afatinib (Phase II)	eGFR and HeR2 inhibitor
AZD5312 (Phase I)	Androgen receptor antisense inhibitor
Everolimus (Phase I) (Phase II)	mTOR inhibitor (mTORC1 and mTORC2)
Rapamycin (Phase I) (Phase II)	mTOR inhibitor
ABi-009 (albumin-bound rapamycin) (Phase I) (Phase II)	mTOR inhibitor
ALT-801 (Phase I) (Phase II)	p53/HLA-A2-expressing tumor cells
HS-410 (Phase I) (Phase II)	immune activator along with BCG
ALT-803 (Phase I) (Phase II)	immune activator through iL-15
Ipilimumab (Phase II)	CTLA-4 antibody
MeDi4736 (Phase I)	PDL1 antibody antagonist
Tremelimumab (Phase I)	CTLA-4antibodyDownregulationofT-reg cells
AGS15e (Phase I)	Slitrk6 targeting immunotherapy
MK-3745 (pembrolizumab) (Phase I) (Phase II)	PDL1
Ad/HeR2/Neu vaccine (Phase I)	vaccination/immune activation
SAR566658 (Phase I)	Anti-CA6-DM4 immunotherapy
Lenalidomide (Phase I)	Immunomodulation
MPDL3280A (Phase I)	Anti-PDL1 immunotherapy
Eribulin mesylate (Phase I) (Phase II)	Microtubule formation/mitosis
Abraxane (Phase I) (Phase II)	Protein-bound paclitaxel—mitosis
Tesetaxel (Phase II)	Tubulin stabilization—antimitotic
ASG-22Ce (Phase I)	Inhibition of tubulin formation in cancer cells by targeting cells expressing adhesion molecule nectin-4 with monomethyl auristatin e
Amrubicin (Phase II)	Anthracycline targeting topoisomerase ii
Gemcitabine	Nucleoside analog targeting S phase
5-Fluoro-2-deoxycytidine with Tetrahydrouridine (Phase II)	inhibition of DNA methylation/cytosine deamination
Romidepsin (Phase I)	HDAC inhibitor
BBi608 (Phase I) (Phase II)	Cancer cell stemness
Ganetespib (Phase I)	inhibition of HSP90
OGX-427 (Phase II)	HSP27 inhibitor
Veliparib (Phase I)	PARP inhibitor
Gefitinib (Phase II)	Inhibit EGFR TKI
Etunimab (Phase II)	Inhibit EGFR TKI
Erlotinib (Phase II)	Blocks EGFR
Trastuzumab (Phase II)	Blocks ErbB2
Lapatinip	Reversed inhibition of EGFR and ErbB2
Sunitinib (Phase II)	Inhibition of VEGFR1–3, PDGFR, C-Kit, and Flt3
Pazopanib (Phase II)	Inhibition of VEGFR1–3, PDGFR, and C-Kit
Sorafenib (Phase II)	Inhibition of VEGFR2/3, PDGFR, Raf, C-Kit, and Flt3
Ad CMV-TP53 (Phase I)	Delivery of functional TP53 into cells
Bevacizymab (Phase II)	Inhibits VEGF antibody
Aflibercept (Phase II)	VEGF binding to endothelial cells and blocking VEGFR interaction
Curcumin	decrease VEGF binding, c-MYC
Sulforaphane	decrease VEGF binding, c-MYC
Resveratrol	decrease c-MYC
Quercetin	decrease VEGF binding, c-MYC

**Table 2:** Breast cancer treatment with a specific targeted drugs.

Target Agent	Process of Intervention
Trastuzumab	Suppresses downstream signaling involved in normal cell proliferation, motility, anti-apoptosis, along with malignant cell invasiveness and angiogenesis
Pertuzumab	Prevents dimerization among HER2 and further HER family members, particularly HER3, and stimulates ADCC (antibody-dependent cellular cytotoxicity), 16 whereas trastuzumab only averts dimerization among HER2 and other HER family members, particularly HER3
Lapatinib	Hinder receptor phosphorylation and inhibit downstream pathways that affect tumor cell proliferation and survival.
T-DM1 (Trastuzumabemtansine)	Transmit the microtubule-inhibitory drug to HER2-positive cancer cells, reducing systemic toxicity along with improving anticancer efficacy
Everolimus	Suppress mTOR activation while also efficiently inhibiting upstream signal transmission, which is important for tumor cell development.
Ipatasertib	Inhibits AKT
Veliparib	PARP1 and PARP2 inhibitors
Talazoparib	Inhibits PARP
Olaparib	PARP inhibitor with potential anticancer efficacy in BRCA1/2-mutated breast cancer patients
Palbociclib, abemaciclib, and ribociclib	CDK4/6 inhibitors
Atezolizummab and durvalumab pembrolizumab	Inhibit the PD-1 receptor-mediated negative immune regulatory signal
Bevacizumab	Inhibits VEGF
Curcumin	regulating p^53^gene expressions
EGCG	upregulation of p21
Genistein	activation of ERK
Lycopene	activation of ERK, Akt, and p70S6 kinases

HER: human epidermal growth factor; PARP: Poly (adenosine diphosphate-ribose) polymerase; CDK: cyclin-dependent kinase; PD: programmed Death; VEGF: vascular endothelial growth factor; AKT: v-akt urine thymoma viral oncogene homolog.

**Table 3:** Drugs used for Kidney cancer and their targets.

Name of Drugs	Targets	Reference
Sorafenib	VEGFR 1–3, C-Kit, PDGFR	[52]
Sunitinib	VEGFR 1–3, C-Kit, PDGFR and Fit-3	[50]
Bevacizumab	VEGF	[50]
Pazopanib	VEGFR 1–3, C-Kit and PDGFR	[53]
Temsirolimus	mTOR	[50]
Everolimus	mTOR	[50]
Axitinib	VEGFR1–3	[50]
Nivolumab	PD1	[50]
Cabozamtinib	MET, RET and VEGFR2	[50]
Lenvatinib	VEGFR1–3, PDGFRβ, RET, FGFR1–4 and KIT	[50]
Regorafenib	VEGFR	[54]
Cediranib	VEGFR 1–3	[55]
Dovitinib	VEGFR and mTOR	[56]
Quercetin	reducing the lipid ROS	[51]
Luteolin	increase p^53^gene and decrease the PUMA-α	[51]
Kaempferol	suppressed TNF-α, activate NF-κB	[51]

**Table 4:** Medications regarding lung cancer therapy.

Drugs	Drugs Mechanism	Tumors Name Which Is Treatable	References
Lirilumab	In CT phase I, halt KIR signaling	Solid-form tumors (Squamous cell carcinoma)	[65]
Paclitaxel	Work as Chemo and immune-cytokines	Melanoma, NSCLC	[66]
Pembrolizumab	Work against programmed death-ligand 1 by reprogramming NK cells.	NSCLC	[67]
Nvolumab	NK cell activation in CT phase II, activity against programmed death-ligand 1.	NSCLC	[65]
Curcumin	inhibiting PI3 K/Akt pathway	-	[64]
β-elemene	inhibiting PI3 K/Akt pathway	-	[64]

**Table 5:** Drugs used to treat melanoma cancer and their targets.

Name of Drugs	Main Target	Reference
Ipilimumab	CTLA-4	[81,82]
Perbrolizumab	PD-1	
Nivolumab	PD-1	
Vemurafenib	BRAF	
Trametinib	MEK and PD-1	
Dabrafenib	BRAF	
Cobimetinib	MEK and PD-1	
Quercetin	inhibit STAT3	[83]
kaempferol	inhibit STAT3	[83]

**Table 6:** Drugs for Prostate cancer and their targets.

Name of Drugs	Targets	References
Bicalutamide	CYP3A4, CYP2C9, CYP2D, CPY2C19, Binding of plasma protein	[97]
Abiraterone	CYP2C8, CYP1A2, CYP3D6	[97]
Enzalutamide	CYP3A4, CTP2C9, CYP2C19, Pgp, BCRP, OATPs	[97]
Abiraterone Acetate	CYP2C8, CYP3D, CYP1A2	[97,98]
Docetaxel	Binding of plasma protein	[87,97,98]
Cabazitaxel	CYP3A4, CYP2C8, BCRP, OATP1B1, OATP1B3, UGT, stabilize tubulin	[97,99]

**Table 7:** Drugs utilized for various types of thyroid cancers along with their target.

Drugs	Drugs Targets	Treatment for Various Cancer Types	References
Axitinib	TKI, VEGFR1–3	ATC, MTC, and DTC	[102,103]
Lenvatinib	TKI, VEGFR1–3, FGFR1–4, RET, PDGFR	ATC, MTC, and DTC	
Cabozantinib	TKI, VEGFR2, MET, FLT3, RET, c-kit	DTC and MTC	
Dabrafenib	STKI, BRAF V600E, MEK1 &2	ATC and DTC	
Everolimus	m-TOR	RCC, TS, SEGA	
Pazopanib	TKI, VEGFR1–3, FGFR1–4, RET, PDGFR, c-kit	ATC, MTC, and DTC	
Larotrectinib	NTRK	NTRK-fused thyroid cancer	
Sorafenib	TKI, VEGFR1–3, FGFR1–4, RET, PDGFR, c-kit, BRAF	ATC, MTC, and DTC	
Sunitinib	TKI, VEGFR1–3, FGFR1–4, RET, PDGFR, c-kit, CSF-1R	DTC and MTC	
Vandetanib	TKI, VEGFR2–3, RET, EGFR	Only MTC	
Vemurafenib	BRAF V600E	Only PTC	

**Table 8:** Drugs, their targets, and cause of Adenoid Cystic Carcinoma.

Name of Drugs	Main Targets of Drugs	Main Pathways of Tumor Formation	References
Everolimus	mTOR	P53, MYB-NF-κB, MYBL1-NFIB fusion, DNA methylation, TGF-β, C-Kit fusion	[110,111]
MK-2206	AKT		
Nelfinavir			
Gefitinib	EGFR		
Sorafenib	PDGFR/VEGFR		
Axitinib			
Dovitinib	FGFR		
Vorinostat	HDAC		

**Table 9:** Natural compounds and targeted cells in cancer treatment.

Sr.No.	Component	Process of Intervention	Target Cell	Reference
1.	Quercetin	Repressed Akt/ERK ½ pathways, inactive NF-κB, COX-2	AMPK α, PI3K-Akt, Akt/ERK ½, NF-κβ, COX-2, ARP-1, RPMI8226	[125]
2.	Andrographolide, 14-deoxyandrographolide	Inactive AKT/REK signaling, dominant MAPK	AKT/REK, MMP2, MAPK, MMP-7, MMP-9, MMP-2	[126,127]
3.	Vinblastine	hinder the PI3K pathways and CDK	PI3K pathways	[128]
4.	β-elemene	resist leukemia (DNR/K562) and GS cell lines (ADR/SGC7901), downregulate Akt signaling pathways, block ABCB1 transporters efflux portion, which over-expressed in KB-C2 cells, resist MCF-7 cells, PTEN expression and Pgp expression	DNR/K562, GS cell lines, ABCB1 transporters efflux portion, MCF-7	[129]
5.	Curcumin	Activation of beta-growth factor (TGF-β), by inhibiting AP 1 and Hypoxia-inducible factors HIF-(1) it stimulate VEGF expression, reduces the cells of MMPs, ICAM-1 and VCAM, increases several anti- metastatic proteins such as, non-metastatic gene NM23, tissue inhibitor metalloproteinase (TIMP 2) and E-cadherin	TGF-β, AP 1, HIF-(1), VEGF, MMPs, ICAM-1, VCAM, NM23, TIMP 2 and E-cadherin	[130,131]
6.	Salinisporamide A	inhibit activation	NF-κB	[132]
7.	Chalcones	activity against HCT116, MCF-7 and 143B cancer cell line	HCT116, MCF-7 and 143B cancer cell line	[133]
8.	Baicalein	Inhibit Akt phosphorylation, activate caspase-3, caspase-9, upregulate Bax expression and downregulate Bcl-2 expression	ERK/p38 MAPK pathway, MCF-7 cell, T24 cells vimentin, N-cadherin, ZEB2, ZEB1, I-kappa-B (IKB)-β, nuclear translocation of p65 and p50	[134]
9.	Neferine	downregulating the PI3K/Akt/mTOR ace-survival signalling pathway, as well as PI3K CIII independent autophagy and ROS-mediated Beclin-1 in human lung A549 adenocarcinoma cells	Bcl2, cycline D1, NF-κB, PI3K/Akt/mTOR signaling, PRAP cleavage, p21, p27, Atg 7, Lc3b, JNK/MAPK protein and Bcl2 protein	[135,136]
10.	9-methoxy ellipticine	Inhibit MCF-7	Cdc25 phosphatase	[137]
11.	Rutin, scopoletin, kaempferol	Reduce the MDA-MB-231cells and MCF-7 cells	MEK and ERK pathways	[138]
12.	Ginsenosides	Inhibition of cell viability and cell apoptosis are encouraged by ginsenoside Rg3 in human ovarian cancer HO8910 cells	HO8910, HCT-116 and SW-480	[139,140]
13.	Aloe-emodin	Reduced hoGG1, hMHT11, and apurinic endonuclease in H460 cells expression	H460 cells, hoGG1, hMHT11	[141]

## Data Availability

Not applicable.
